# Co-structure analysis and genetic associations reveal insights into pinworms (*Trypanoxyuris*) and primates (*Alouatta palliata*) microevolutionary dynamics

**DOI:** 10.1186/s12862-021-01924-4

**Published:** 2021-10-20

**Authors:** Brenda Solórzano-García, Ella Vázquez-Domínguez, Gerardo Pérez-Ponce de León, Daniel Piñero

**Affiliations:** 1 Departamento de Ecología Evolutiva, Instituto de Ecología, Universidad Nacional Autónoma de México, 04510 Mexico City, Mexico; 2 Departamento de Ecología de la Biodiversidad, Instituto de Ecología, Universidad Nacional Autónoma de México, 04510 Mexico City, Mexico; 3 Instituto de Biología, Universidad Nacional Autónoma de México, 04510 Mexico City, Mexico; 4 Present Address: Departamento de Sistemas y Procesos Naturales, Escuela Nacional de Estudios Superiores - Merida, Universidad Nacional Autónoma de México, Yucatán, Mexico

**Keywords:** Coevolution, Ecological interactions, Gene flow, Host–parasite associations, Host-specificity, Parasitism

## Abstract

**Background:**

In parasitism arm race processes and red queen dynamics between host and parasites reciprocally mold many aspects of their genetics and evolution. We performed a parallel assessment of population genetics and demography of two species of pinworms with different degrees of host specificity (*Trypanoxyuris multilabiatus*, species-specific; and *T. minutus,* genus-specific) and their host, the mantled howler monkey (*Alouatta palliata*), based on mitochondrial DNA sequences and microsatellite loci (these only for the host). Given that pinworms and primates have a close co-evolutionary history, covariation in several genetic aspects of their populations is expected.

**Results:**

Mitochondrial DNA revealed two genetic clusters (West and East) in both pinworm species and howler monkeys, although population structure and genetic differentiation were stronger in the host, while genetic diversity was higher in pinworms than howler populations. Co-divergence tests showed no congruence between host and parasite phylogenies; nonetheless, a significant correlation was found between both pinworms and *A. palliata* genetic pairwise distances suggesting that the parasites’ gene flow is mediated by the host dispersal. Moreover, the parasite most infective and the host most susceptible haplotypes were also the most frequent, whereas the less divergent haplotypes tended to be either more infective (for pinworms) or more susceptible (for howlers). Finally, a positive correlation was found between pairwise p-distance of host haplotypes and that of their associated pinworm haplotypes.

**Conclusion:**

The genetic configuration of pinworm populations appears to be molded by their own demography and life history traits in conjunction with the biology and evolutionary history of their hosts, including host genetic variation, social interactions, dispersal and biogeography. Similarity in patterns of genetic structure, differentiation and diversity is higher between howler monkeys and *T. multilabiatus* in comparison with *T. minutus*, highlighting the role of host-specificity in coevolving processes. *Trypanoxyuris minutus* exhibits genetic specificity towards the most frequent host haplotype as well as geographic specificity. Results suggest signals of potential local adaptation in pinworms and further support the notion of correlated evolution between pinworms and their primate hosts.

**Supplementary Information:**

The online version contains supplementary material available at 10.1186/s12862-021-01924-4.

## Background

Ecological interactions drive evolutionary change, where each member of the association acts as a natural selective agent to its counterpart. Coevolutionary changes occurring among participants will be more or less evident depending on the strength, frequency and dependency of the interaction. Parasitism constitutes an intimate association in which arm race processes and red queen dynamics between host and parasites reciprocally mold many aspects of their genetics, physiology, morphology, behaviour, and life history traits [1–3]. Genetic studies about host–parasite systems have documented how the life cycle of the parasite and the degree of host specificity, jointly with host population size and dispersal capability, are key factors influencing the genetic structure of parasites and the potential to form coevolutionary associations [2, 4–8]. For instance, a study with bats and their parasitic mite showed a tight link between the genetic structure of the parasite and its host’s social structure [9]. Also, the level of agreement between the genetic patterns of host and parasite were related to the level of host specificity in the Galapagos hawk and three ectoparasites species, in which the highly specific louse (*Degeeriella regalis*) showed congruent genetic structure with the hawk, yielding insights about the host’s recent evolutionary history [10]. Additionally, gene flow in parasites with complex life cycles can be markedly influenced by the dispersal of the most vagile host [11], while strong genetic drift has been observed in parasite populations whose hosts have low dispersal abilities and small home ranges [12]. Correlations between genetic distances of host and parasite have also been observed in parasites with complex life cycles involving free-living stages, like *Schistosoma mansoni* and its definitive rat host [11], and between the freshwater New Zealand snail (*Potamopyrgus antipodarum*) and its trematode parasite (*Microphallus* sp.) [13]. Furthermore, a cophylogenetic study evaluating the evolutionary histories of mammal hosts and helminth parasites showed that the host’s phylogenetic history is a key driver of host–parasite associations and parasite cross-species transmission potential [14].

Although evolutionary interactions between host and parasites can be tight enough to yield correlated genetic patterns and even cophylogenetic relationships and cospeciation [14–16], concordance between host and parasite microevolution is not always straightforward, where asynchronous coevolutionary dynamics can arise, promoting either local adaptation or maladaptation [17, 18]. Commonly, parasites are expected to be more locally adapted than their hosts, exhibiting higher mean performance in “home” hosts than in “away” hosts [19]. Uneven dispersal rates between hosts and parasites are likely to disrupt local adaptation processes [5, 20], resulting in differing degrees of susceptibility/infectivity among host and parasite populations. Hence, parallel assessments of the evolutionary history and population genetics of host and parasites are essential for the understanding of local adaptation and host specificity, the evolution of virulence and host resistance, as well as the emergence of evolutionary associations in which both host and parasite successfully coexist.

Studies about genetic relationships, divergence and coevolutionary patterns between non-human primates and their parasites have evaluated parasite host-specificity and parasite diversification regarding host phylogeny, predominantly with infectious disease agents and some ectoparasites [21–25]. Here we explore the synchrony of microevolutionary dynamics between a metazoan parasite and its host, for which pinworms and non-human primates represent a most suitable study system. Pinworms are parasitic nematodes with direct life cycle and no free-living stage, their eggs survive only a few days once released to the environment, and transmission occurs mainly by direct contact [26]. These features make pinworms highly dependent on host movement for dispersion among host populations. Moreover, pinworms are host-specific parasites showing a close coevolutionary history with their primate hosts, supported by cophylogenetic studies [27, 28] and by correlations between parasite–host life history traits, including pinworm body size and primate longevity and immune responses [29, 30]. Consequently, one might expect covariation of diverse genetic attributes such as diversity and differentiation between pinworms and primate populations.

Mantled howler monkeys (*Alouatta palliata*) are endangered primates, whose arboreal nature and predominant folivorous diet significantly limit their dispersal capability across an unforested matrix [31]. This primate species is distributed from western Ecuador and northern Colombia to southeastern Mexico, where Mexican howler monkeys represent the northernmost distribution of primates in the American continent [32]. As a result of intense habitat fragmentation throughout their distribution range in southeast Mexico, most of their populations are isolated in forest remnants surrounded by anthropic land use [33]. *Alouatta palliata* is parasitized by two species of pinworms, *Trypanoxyuris minutus* which is widely dispersed, found in several howler monkey species including *A. belzebul*,* A. caraya*, *A. guariba*, *A. pigra*, and *A. seniculus* [34]; and *T. multilabiatus*, which has only been reported in *A. palliata* [35]. Both parasites are highly prevalent in mantled howler populations in Mexico and mixed infections are common [36].

We analysed the genetic diversity, genetic structure and demographic history of *A. palliata* and their pinworms from across its geographic range in southeast Mexico, using mitochondrial DNA (mtDNA) for both host and parasites along with microsatellite data only for the host. Given the biology and direct mode of transmission of these parasites, with no vectors or intermediary hosts that could influence parasite genetic configuration other than howler monkeys, the close evolutionary association between pinworms and primates, in conjunction with the likely higher evolutionary potential of the parasite compared to that of their host, we predict: (1) higher genetic diversity and stronger genetic structure in pinworms in comparison with howler groups, given that the parasite’s larger populations sizes and shorter generation times render the effects on genetic patterns of habitat fragmentation and limited host dispersal more quickly detectable in parasites in comparison with the host; (2) a positive association between genetic distances of host and parasites, indicating that genetically similar host populations harbour similar parasite populations, thus implying a dependence of the pinworms gene flow on primate movement; (3) concordant genealogical patterns between host and parasites; and (4) signs of local adaptation in the pinworm species; the last two predictions are associated with both the host-specificity and the coevolutionary hypothesis for pinworms and primates. In order to address this last point, we assessed host–parasite mtDNA haplotype relationships and evaluated how these associations relate to haplotype divergence, parasite infectivity and host susceptibility.

## Results

### Genetic structure, differentiation and diversity in host and parasites

**Fig. 1:**
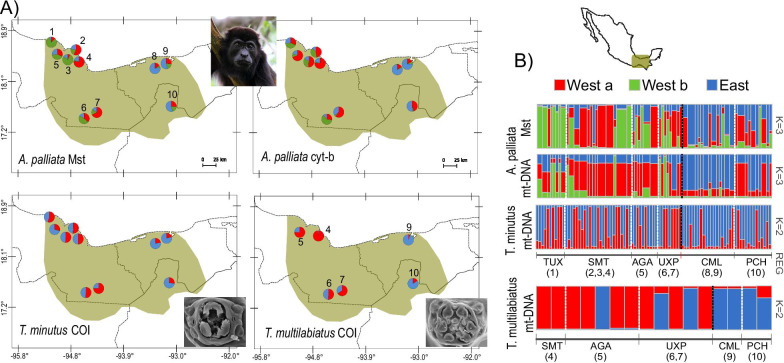
Study site and population genetic structure of *Alouatta palliata* and its pinworms *Trypanoxyuris minutus* and *T. multilabiatus*. **A** Maps showing sampling localities for host and the two parasite species; pie charts depict average per cluster assignment values in each population. **B** Barplots of ancestry proportions (Structure results), based on mitochondrial cytochrome *b* (cyt*-b*) and microsatellites loci (Mst) for the host and on mitochondrial cytochrome oxidase subunit 1 gene (*COI*) for the parasites, in the six studied regions: TUX = Los Tuxtlas (1. Montepio), SMT = Santa Marta (2. Playa, 3. Mirador Pilapa, 4. La Valentina), AGA = Agaltepec island (5), UXP = Uxpanapa (6. Plan de Arroyo, 7. Murillo Vidal), CML = Comalcalco (8. Hacienda la Luz, 9. Archaeological Site), PCH = Pichucalco (10); numbers correspond to locations in figure **A**

Structure results revealed two main genetic clusters corresponding to West and East sampling localities in both pinworm species and the howler monkey, although the clustering is stronger in the host (Fig. 1). The West cluster comprises populations from Los Tuxtlas, Santa Marta and Uxpanapa regions, whereas the East cluster includes the Comalcalco and Pichucalco regions. Individuals from Agaltepec island (the howler semi-captive population) were assigned to the West cluster for *A. palliata* and *T. multilabiatus*, but to the East cluster for *T. minutus.* A third cluster was evident only for howler monkeys (mtDNA and microsatellites), further dividing western localities into two genetic clusters (West a and b; Fig. 1). The AMOVA results showed that genetic variability is distributed among clusters, with similar values in howler monkeys (*F*_*CT*_ = 0.256, p < 0.001) and *T. multilabiatus* (*F*_*CT*_ = 0.277, p = 0.07), while smaller in *T. minutus* (*F*_*CT*_ = 0.074, p = 0.005)*.* Pairwise *F*_*ST*_ differentiation was significant between all regions for howler monkeys and between West and East clusters for *T. minutus*, whereas *T. multilabiatus* exhibited no significant differentiation (Additional file 1: Tables S1 and S2). At a local scale, significant pairwise *F*_*ST*_ values between sampling localities within the same region were observed only for howler monkeys (Additional file 1: Table S3). Isolation by distance (mtDNA Mantel tests) was observed for both pinworm species, while howler monkeys only showed significant isolation by distance based on microsatellites *R*_*ST*_ (Additional file 1: Fig. S1).

**Fig. 2:**
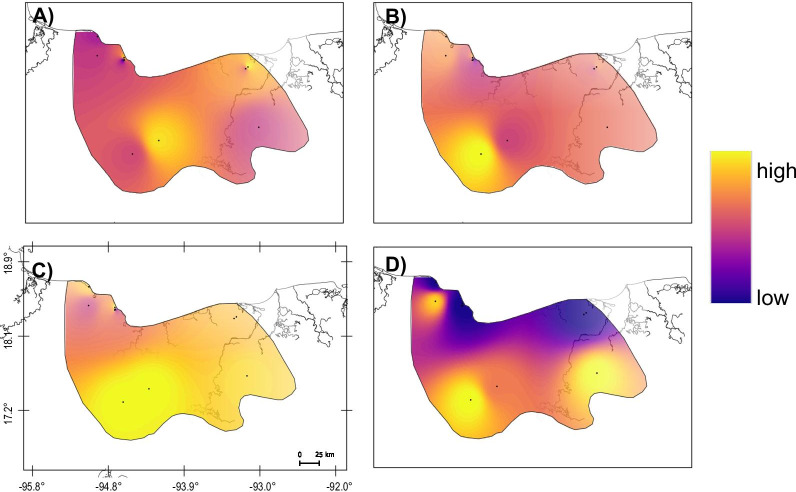
Interpolation maps showing the distribution of genetic diversity in the host and the two pinworm species across their range in Mexico. **A** *Alouatta palliata* expected heterozygosity from microsatellite data; **B** *A. palliata* haplotype diversity (*Hd*) from cyt*-b* sequences; **C** *Trypanoxyuris minutus* and **D** *T. multilabiatus* haplotype diversity (*Hd*) from *COI* sequence data. Black dots represent sampling localities

Genetic variability based on mtDNA showed higher haplotype and nucleotide diversity in the two pinworms species in comparison with the host (Additional file 1: Table S4). In all cases, the West cluster had higher mtDNA diversity values than the East cluster; instead, no differences were observed for the nuclear diversity in the host. Genetic diversity is distinctly distributed in each species as shown in the interpolation maps, although certain similarities can be identified with mtDNA (Fig. 2). Overall, eastern groups tend to be less genetically diverse compared to western ones, except for Santa Marta region (SMT) that has markedly lower genetic diversity in *T. multilabiatus*.

### Correlation between genetic distances of host and parasites

A significant positive correlation was found between *T. minutus* and howler monkeys *F*_*ST*_ pairwise distances (*r* = 0.53*, p* = 0.008); all other computed genetic distances showed no correlation (*D*-Jost: *r* = 0.12*, p* = 0.29*;* Hedrick’s *G*_ST_: *r* = 0.11, *p* = 0.29; Edwards: *r* = 0.04, *p* = 0.4) (Additional file 1: Fig. S2). For *T. multilabiatus* and howler monkeys*,* a positive correlation was found between pairwise Hedrick’s *G*_ST_ (*r* = 0.64*, p* = 0.02) and *F*_*ST*_ (*r* = 0.75*, p* = 0.02)*; D*-Jost (*r* = 0.08 *p* = 0.42) and Edward distances (*r* = 0.07*, p* = 0.5) were not significant (Additional file 1: Fig. S2).

### Demographic history and genealogies

**Fig. 3:**
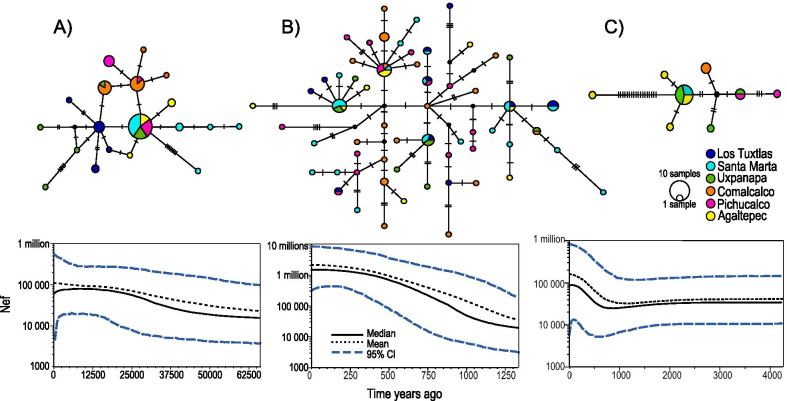
Haplotype genealogical relationships and demographic history of **A** *Alouatta palliata*, **B** *Trypanoxyuris minutus* and **C** *T. multilabiatus*. Top: median-joining haplotype networks, colours correspond to sampled geographic regions in southeast Mexico. Bottom: Bayesian skyline plots based on mtDNA showing changes in median female effective population sizes (Nef) through time. **A** Gradual population growth in howler monkeys until ca. 8000 years ago, decreasing afterwards until reaching a most recent Nef of 60,000. **B** Continuous population growth in *T. minutus* until ca. 250 years ago when the increase rate slowed down to a relatively constant trend (Nef from 1,303,500 to 1,347,000. **C** dynamic trend in *T. multilabiatus* population growth, remaining constant until ca. 2000 years ago and then fluctuating by decreasing from 32,250 to 24,700, followed by a rapid increase around 800 years ago up to 88,400, to a final decrease with a most recent Nef of 86,000

The Bayesian skyline plots (BSPs) results supported larger population sizes in parasites compared with those of their host, exhibiting particular demographic histories for each species. The howler monkey showed a gradual population growth and recent population decline. *Trypanoxyuris minutus* also showed a past continuous population growth which seems to slow down more recently, while a dynamic behaviour with a decreasing and final increase trend towards the present was observed in *T. multilabiatus* (Fig. 3).

Regarding the haplotype evolutionary history, 19 different haplotypes were found in howler monkeys, 59 in *T. minutus* and 8 in *T. multilabiatus*. The host median-joining network differed in several aspects from that of the pinworms. First, a most frequent haplotype, likely ancestral, in the howler monkey network, present across all regions except TUX and CML (Fig. 3a). Instead, the *T. minutus* genealogy resulted in a complex network with many alternative paths between haplotypes and no geographic concordance; the most parsimonious tree showed few frequent haplotypes and many singletons, most of them specific to certain localities (Fig. 3b). Also, the howler monkey haplotypes were connected by 1 to 8 mutational steps, while haplotypes of the pinworms showed shorter connections (1 to 3 mutational steps), except for one *T. multilabiatus* haplotype separated from the rest by 18 mutational steps (Fig. 3c). The codivergence test showed no significant congruence between host and parasite phylogenies (global test = 0.00014, *p* = 0.854), suggesting random evolutionary associations between howler monkey and *T. minutus* haplotypes.

### Host susceptibility, parasite infectivity and haplotypes associations

**Fig. 4:**
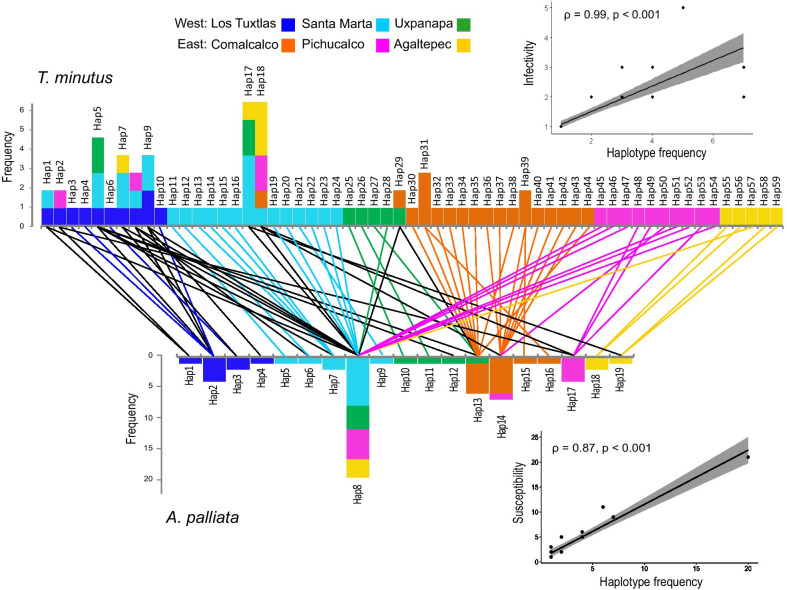
Host/parasite haplotype associations between howler monkeys (*Alouatta palliata*) and pinworms (*Trypanoxyuris minutus*). Bars represent haplotype frequency and lines indicate the associations among haplotypes. Colours correspond to geographic regions. Black lines depict associations occurring in different regions. Top inset: Spearman correlation between *T. minutus* haplotype frequency and haplotype infectivity (number of different host haplotypes associated to each pinworm haplotype). Bottom inset: Spearman correlation between *A. palliata* haplotype frequency and vulnerability (number of different pinworm haplotypes co-occurring within each host haplotype)

The associations between howler and *T. minutus* haplotypes along with their frequency are shown in Fig. 4. Eighty one percent of the *T. minutus* haplotypes (48/59) were associated to single host haplotypes, most of them specific to certain localities, whereas only 11 pinworm haplotypes infected 2 or more host haplotypes, with a mean of 1.3 host haplotypes infected by each pinworm haplotype. Haplotype Hap5 was the most infective, parasitizing five different howler haplotypes. A positive correlation was observed between infectivity and frequency, with the most infective *T. minutus* haplotypes also being the most frequent (ρ = 0.99, *p* < 0.001; Fig. 4). Regarding the host, each howler haplotype was infected by a mean of 4 different pinworm haplotypes (1–21), where the most frequent haplotypes were also the most susceptible (ρ = 0.87, *p* < 0.001; Fig. 4).

**Fig. 5:**
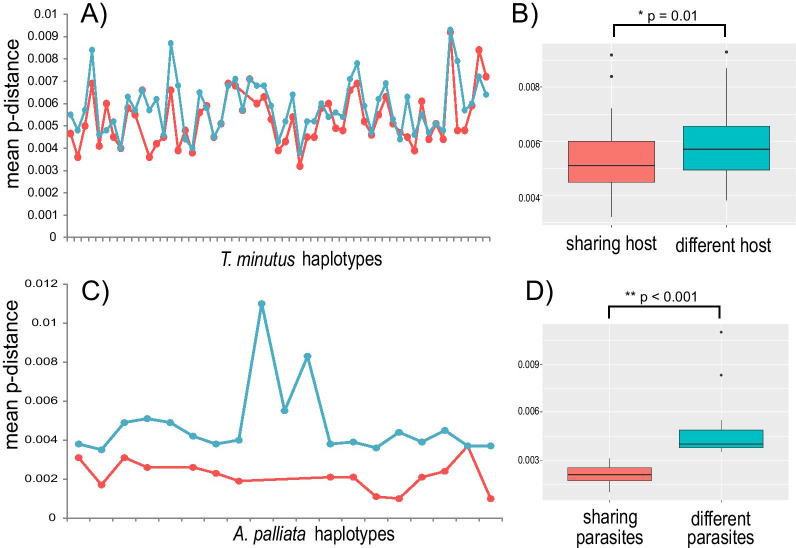
Mean genetic distance between haplotypes sharing host/parasite and those associated to different host/parasite haplotypes. **A** Line graphs showing the mean p-distance values between sharing (red) and differing host (light blue) for each *Trypanoxyuris minutus* haplotype. **B** Boxplot showing the differences on p-distance between pinworm haplotypes sharing and differing host haplotypes. **C** Line graphs showing the mean p-distance values between sharing and differing parasites for each *Alouatta palliata* haplotype. **D** Boxplot of the differences on p-distance between host haplotypes sharing and differing pinworm haplotypes. *P* values derived from Wilcoxon–Mann Whitney test

A negative correlation was found between haplotype infectivity/susceptibility and mean haplotype p-distance, where less divergent haplotypes tend to be either more infective (pinworm haplotypes associated to a larger number of host haplotypes) (τ = 0.32, *p* = 0.002), or more susceptible (howler haplotypes parasitized by a larger number of pinworm haplotypes) (τ = 0.63, *p* < 0.001; Additional file 1: Fig. S3). When comparing haplotypes that share host/parasite, p-distance is lower between pinworm haplotypes that parasitize the same host haplotype than those infecting different host haplotypes (Fig. 5a, b). The same occurred for howler monkeys, p-distance between haplotypes sharing pinworm haplotypes was lower than those parasitized by different *T. minutus* haplotypes (Fig. 5c, d). Finally, a positive correlation was found between host haplotypes pairwise p-distance and the genetic distance of their associated pinworm haplotypes, where similar host haplotypes tend to harbour genetically similar pinworm haplotypes (Additional file 1: Fig. S4).

## Discussion

Here we present a co-structure analysis of the microevolutionary dynamics of the coevolving system between the mantled howler monkey *Alouatta palliata* and its two parasitic pinworms, differing in their degree of host specificity across the host’s distribution range in southeastern Mexico. The genetic and demographic patterns we observed support the notion of correlated evolution between pinworms and their primate host.

### Host–parasite genetic patterns and microevolutionary dynamics

The patterns of genetic structure, differentiation and diversity are more similar between howler monkeys and its host species-specific pinworm *Trypanoxyuris multilabiatus* than with *T. minutus*, the host genus-specific. These findings support the tight evolutionary association previously suggested in a phylogenetic study where divergence in *T. multilabiatus* follows mantled howler monkey subspecies, while in *T. minutus* this pattern is absent [37].

As predicted, genetic diversity was higher in both pinworm species compared to that of the howler monkey. Although pinworms exhibit a haplodiploid mode of reproduction [26], the high genetic diversity observed in this and previous studies [38] suggests that sexual reproduction in *Trypanoxyuris* might be more frequent than asexual. Another alternative would be that, despite having a predominantly asexual reproduction, the high genetic variability might result from the large parasite population sizes. Notably, genetic variation was also higher in *T. minutus* than in *T. multilabiatus*, which can be explained by the relationship between genetic diversity and effective population size [39], given that *T. multilabiatus* is markedly less abundant and with an apparent restricted distribution—it has not been found in the northernmost mantled howler populations of Los Tuxtlas [35]. Greater genetic diversity can also be related to the degree of host specificity; that is, a broader host spectrum in *T. minutus* could trigger not only larger population sizes but also the need to adapt to different host environments, hence higher genetic variation. We acknowledge further evaluation is needed because in this study the *T. multilabiatus* sample size was small.

We predicted higher differentiation in the parasites due to their dependent and more restricted migration. However, despite the presence of two genetic clusters (West and East) in both parasites and their host, genetic structure and genetic differentiation, contrary to our prediction, were stronger in the host than in both pinworms species, in agreement with limited dispersal in the primate as previously documented for this species [40–42]. Mazé-Guilmo et al. [5] suggest that variables related to host dispersal could be poor predictors of genetic patterns in parasites, and that alternative factors like the host’s and the parasite’s biology are also key drivers of the codistribution of their genetic variation. Nonetheless, in parasites with direct life cycles lacking free-living stages, as is the case in pinworms, host and parasite concordant pairwise genetic differentiation might be expected [5]. We observed a positive correlation between genetic distances of howler monkeys and both pinworm species which indicates that genetically similar host populations harbour genetically similar parasites, suggesting that pinworm gene flow is mediated by howler monkey dispersal. Large population sizes in these parasites, as evidenced by the demographic (BSP) results, might be counteracting the effects of genetic drift, while higher gene flow in the parasites can also contribute to their lower genetic structure. Given that one howler individual can harbour a large number of pinworms (up to ~ 62,000 adult pinworms have been counted in a howler monkey individual [43]), the dispersal of just one monkey could signify a gene flow many times higher in magnitude among pinworms populations compared to that of primates, explaining the parasites’ higher gene flow despite its dependency on host movement. Moreover, certain host behaviour such as prospecting movements can favour the dispersal of infection agents among host populations without necessarily involving host genetic interchange [44], rendering the correlation between host and parasite gene flow less straightforward.

While an isolation by distance (IBD) pattern was observed for the pinworms based on mitochondrial data, for the howler monkey it was identified only with nuclear data. The latter can be related to the historical dispersal of howler monkeys, whereas the local genetic differentiation and IBD observed with the genetically more variable microsatellites loci could reflect a contemporary constraint in individual movement. The significant habitat loss and landscape transformation derived from human activities along the primate distribution has most likely limited host dispersal between closer populations. Furthermore, IBD in pinworms implies greater potential for parasite transmission between adjacent howler monkey populations due to spatial proximity that increases the contact rates among host individuals. Indeed, inter-host contact and proximity, animal movement and spatial constrains imposed by a heterogenous landscape, all play a critical role in parasite transmission dynamics in wildlife populations [45–48]. For instance, the extent of physical contact between and within social groups has major implications in primate epidemiology, easing the spread of pathogens and parasites [49–51]. In fact, higher *Trypanoxyuris* infection in howler monkeys has been associated to closer partner proximity [52].

The overall demographic and genetic architecture of both pinworm species (high genetic diversity, high gene flow, shorter generation time and large effective population sizes) suggest higher evolutionary rates in the parasites compared to their host. This is evident in the haplotype genealogies where pinworms showed complex networks formed by many unique haplotypes differing by few mutational steps, compared to the simple and more structured haplotype network in the howler monkey. Even though cophylogenetic patterns between pinworms and their primate hosts have been documented at macroevolutionary scales [27, 28, 37], the intraspecific analyses we performed did not detect congruent divergence among pinworms and howlers, suggesting instead distinct diversification processes. Disparate rates of molecular evolution have been documented in a host-specific and coevolving host–parasite system (e.g. pocket gophers and their chewing lice ectoparasites), with differences in mutational rates and generation times as the most plausible mechanisms accounting for the rate disparities [53]. Considering that we sampled populations along the northernmost portion of the *Alouatta palliata* howler monkeys distribution (complete geographic range encompassing from western Ecuador and northern Colombia to southeast Mexico [32]), the higher rate of evolutionary change in the parasite could be impeding the detection of codivergent pinworm-howler patterns at this narrow spatial scale. We predict this codivergent pattern to be more evident at broader geographical scales (i.e. the entire host distribution), where both host and parasites have had a longer time to accumulate genetic differences.

### Genetic variants, infectivity, susceptibility and geography

We aimed to further explore the genetic association between pinworm and howler monkeys by using mtDNA haplotype identity to assign genetic variants to host and parasite individuals, enabling us to describe susceptibility and infectivity traits based on the number of host–parasite connections identified per haplotype. Accordingly, hosts were considered more susceptible if they were parasitized by a greater number (diversity) of pinworm haplotypes, whereas pinworms infectivity was defined by the number of different host haplotypes in which each pinworm was found.

Overall, nearly each howler haplotype was parasitized by more than one *T. minutus* genetic variant. Conversely, only few pinworm haplotypes were found parasitizing more than one host genetic variant, and most *T. minutus* were associated to a single howler haplotype, suggesting that *T. minutus* tends to adapt to one host genetic configuration. Selection can cause parasites to develop genetic specificity towards a particular host genotype, usually the most common, increasing its susceptibility [19, 39]. Our results agree, where the most frequent howler/pinworm haplotype was also the most susceptible/infective. This genetic specificity could explain the frequent one-to-one association between *T. minutus* and the howlers’ genetic variants, as well as the higher genetic similarities between pinworm/host haplotypes that share hosts/pinworms haplotypes, supporting that genetic similarity among hosts might be a key factor for pinworm transmission and establishment. Additionally, these genetic associations could be related with the parasite transmission mode, where autoinfection and retroinfection are common mechanisms for pinworm acquisition [54, 55]. Both mechanisms of transmission allow several generations of pinworms of the same genetic pool to continue infecting that individual host. This could hinder the spread of pinworm variants among host individuals within the population, facilitating the development of specificity.

Our study also reveals that parasite genetic variants are not evenly distributed across geographic regions, and that only some pinworm haplotypes could be considered highly infective (being present in high frequency in all studied localities). Additionally, host haplotypes unique to a particular geographic region tend to harbour pinworm variants also unique for that region. This geographic specificity [56] in *T. minutus* shows a roughly northwest to southeast gradient where many of the northernmost pinworm haplotypes of Los Tuxtlas are also present in other regions. Comparatively, most of the southeastern haplotypes (Comalcalco and Pichucalco) are only found in that particular region. The history of dispersion of howler monkeys across southern Mexico helps explain this pattern, which followed a colonization process from south to north [57], thus the northernmost populations are the most recent [42]. During such range expansion events, parasites can either travel with their host into new locations or never reach the newly established populations because they were lost in the process or because the migrants did not carry the parasite with them. The increasing northwest to southeast specificity gradient observed in *T. minutus* suggests that howler monkeys carried most of the pinworm genetic variants as they dispersed towards northern regions. Also, that only a fraction of pinworm haplotypes remained within the already established host populations. Therefore, most recent host populations in the north still harbour a collection of pinworm mitochondrial geographic variation.

Notably, the relationships between haplotype divergence and parasite infectivity and host susceptibility, jointly with the parasite’s higher gene flow, suggest potential local adaptation in pinworms. Local adaptation, a higher mean fitness of populations in local environments, is linked to the ability of each organism to incorporate new genetic and phenotypic variants that can confer some fitness advantage [20]. Host–parasite systems induce constant evolutionary change in order to overcome the selective pressures imposed by this antagonistic interaction. When parasites show higher evolutionary rates and higher gene flow than their hosts, they are expected to be locally adapted, performing better in sympatric or home hosts than in allopatric or away hosts [19, 58]. If we translate this into genetic terms, we expect locally adapted parasites to be more infective to genetically similar hosts than to genetically different hosts (see [59]). Indeed, we found that more divergent hosts were less susceptible to be parasitized by different pinworms haplotypes, suggesting higher performance of the parasite (infectivity) in genetically similar hosts compared to differing ones. Considering that mtDNA divergence reflects the accumulation of genetic differentiation along the host and parasite historical associations, the genetic variation observed likely echoes an advantage for the host but not so much for the parasite, since most divergent *T. minutus* haplotypes were less infective (associated to a smaller number of host haplotypes). Although acquiring genetic variation could favour parasite infectivity, it is suggested that above certain threshold it could also increase host resistance [20, 60]. Fluctuations between host–parasite migration and mutation rates causes cycle oscillations of infectivity and resistance via frequency dependent selection, and this may play a key role in local adaptation and maladaptation dynamics, which in turn are fundamental for host-parasite coevolution [18, 20, 61]. Hence, the fact that similar howler monkey haplotypes harbour genetically similar *T. minutus* infrapopulations, further suggests a correlated evolution in agreement with a highly host-specific and evolutionary intimate system, as shown by these pinworms and primate.

Parallel studies on host–parasite genetic arrangements, although challenging, are growing attention and interest. Our study, jointly with examples (as those mentioned along the text) that encompass systems with different degrees of host specificity and distinct parasite life cycles and life history strategies, contribute to understanding the transmission dynamics, the distribution of resistant and virulence alleles, and the spread of disease. Also enabling a better comprehension of the coevolutionary process and their role in preserving genetic variation, namely the persistence of host and parasite populations. Furthermore, the use of conventional genetic techniques, like in our study, has been of enormous value to uncover relevant information about the evolutionary ecology of hosts and parasites interactions. Incorporating high throughput sequencing techniques and sampling across the whole genome could certainly be of great value in co-structure studies, by yielding detailed information on the microevolutionary changes in pinworms and their primate hosts, fostering a thorough understanding of the genetic, ecological and evolutionary dynamics between hosts and parasites [62].

## Conclusions

Evolutionary processes of mantled howler monkey populations and their pinworms are indeed tightly linked. Our mtDNA findings show that pinworm gene flow is mediated by host dispersal, while at the same time no codivergence was observed between pinworms and their primate host. The high genetic diversity, high gene flow and large effective population sizes showed by the two pinworm species indicate higher evolutionary rates in the parasites compared to their host. Additionally, genetic structure, differentiation and diversity patterns show higher similarity between howler monkeys and the host species-specific pinworm *T. multilabiatus* than the host genus-specific *T. minutus*, highlighting the role of host-specificity in coevolving processes. Our findings show that pinworms are more infective in the genetically similar host, whereas associations of host and parasite genetic variants reveal both genetic specificity towards the most frequent host haplotype and geographic specificity in *T. minutus*. Moreover, altogether these results suggest signals of local adaptation in the parasite, while the fact that similar howler monkey haplotypes harbour genetically similar *T. minutus* infrapopulations further supports the notion of correlated evolution between pinworms and their primate hosts.

## Methods

### Data collection

We sampled free-ranging mantled howler monkey groups (*Alouatta palliata*) and their pinworms at ten sampling localities across six geographic regions in southeast Mexico, using non-invasive techniques (Fig. 1). One locality, Agaltepec island, harbours a semi-captive population of howlers (AGA; Fig. 1). We collected howler faecal samples right after deposition and placed them in 50 ml tubes with 100% ethanol. Before storing, we performed a macroscopic examination of each faecal sample searching for adult pinworms, which were removed with a fine paint brush and placed in 1.5 ml tubes with 100% ethanol. A total of 105 pinworms (89 *Trypanoxyuris minutus* and 16 *T. multilabiatus*) were recovered from 58 howler monkey individuals. Pinworm specimens were labelled with host ID to be able to link host and parasite DNA. Host samples and pinworm specimens were stored at − 20 °C until DNA processing.

### Host and parasite genetic data

Howler monkey DNA was extracted using the Norgen stool DNA isolation kit following manufacturer’s instructions. For each individual host, we amplified a 964 pb fragment from the mitochondrial cytochrome *b* gene (cyt*-b*) and genotyped nine microsatellite markers. Details about host DNA amplification and genotyping (cyt-*b* and microsatellites loci) procedures are found in Solórzano-García et al. [42]. For parasite DNA, individual pinworms were digested overnight at 56 °C in a solution containing 10 mM Tris–HCl (pH 7.6), 20 mM NaCl, 100 mM EDTA (pH 8.0), 1% Sarkosyl, and 0.1 mg/ml proteinase K. DNA was extracted from the supernatant using the DNAzol^®^ reagent (Molecular Research Center, Cincinnati, OH) according to the manufacturer’s instructions. A fragment of ~ 800 bp of the mitochondrial cytochrome oxidase subunit 1 gene (*COI*) was obtained for each recovered pinworm of both species. For details on pinworm DNA amplification procedures see Solórzano-García et al. [38]. Mitochondrial DNA (mtDNA) alignments were built using Clustal Omega [63] via the EMBL-EBI web interface [64]. As an additional accuracy assessment, sequences were translated into amino acids using MESQUITE v.3.2 [65] with the corresponding vertebrate or invertebrate mitochondrial genetic code to check for the presence of stop codons. Both primate host and parasites mtDNA sequences are available in GenBank; associated microsatellite genotypes of primate host are available at https://doi.org/10.5281/zenodo.4538731 (Additional file 1: Table S5).

### Comparison of host and parasite genetic structure

We assessed genetic structure in host and parasites with Structure v.2.3.4 [66], by testing clusters (*K*) from 1 to 6, running 20 replicates per *K* of 1,000,000 MCMC and 100,000 iterations as burnin under the admixture model. The most probable number of clusters was estimated using the Evanno method [67]; Structure results were visualized using Pophelper v.2.3 in R [68]. Additionally, an analysis of molecular variance (AMOVA) was performed in Arlequin v.3.5.2.2 [69] to assess the level of genetic differentiation among clusters, geographic regions and sampling localities within regions, for host and parasite mtDNA.

Because howler monkey movements are expected to be higher among groups located in nearby forest fragments, and dispersing individuals may carry pinworms with them, we tested the isolation by distance hypothesis (IBD) for the host and the two pinworm species with Mantel tests, comparing genetic (mtDNA) and geographic distances. We estimated genetic distances between sampling localities based on *D*-Jost [70], Hedrick’s *G*_ST_ [71], and Edwards [72] with the package mmod v.1.3.3 [73] for host and parasites; we also estimated conventional *F*_ST_ with Arlequin. Geographic Euclidian distances between sampling localities were calculated with Raster v.3.3 [74]. IBD patterns were also tested for host microsatellite data based on *R*_*ST*_ and estimated in Arlequin. Mantel tests were run with vegan v.2.5 [75] and IBD correlation plots were built with MASS v.7.3 [76]. Next, in order to test the hypothesis that parasite gene flow is mediated by host dispersal, we examined the correlation between mtDNA genetic distances of howler monkey populations and those of their pinworms. Considering that pinworms are directly transmitted host specific parasites, we would expect dispersal of the parasite to be determined by that of the host, hence two groups of howlers connected by migration should harbour genetically similar pinworms.

### Distribution of genetic diversity

Molecular diversity indices including the number of segregating sites (*S*), haplotype diversity (*Hd*) and nucleotide diversity (π) were obtained with DnaSP v.5 [77] for howler monkey and pinworms per sampling locality and genetic cluster (see “Results”). In addition, genetic diversity estimates for host microsatellite data were estimated as the average number of alleles (*Na*) and observed (*Ho*) and expected heterozygosity (*He*) with Arlequin. The geographic distribution of genetic diversity was explored by mapping the haplotype diversity of both host and parasites, as well as the host expected heterozygosity per sampling locality. We applied the Inverse Distance Weighted interpolation method (IDW) to spatially interpolate genetic diversity values between sampling localities across the distribution of *Alouatta palliata* in Mexico, using the Quantum Geographic Information System QGIS 3.14.16. The resultant raster map was clipped according to the host distribution polygon [78].

### Demographic history and genealogical analysis

We examined changes in population sizes over time by constructing a Bayesian Skyline Plot (BSP) in BEAST v.1.7.5 [79] for each pinworm species and howler monkeys. BSP analyses were run under the strict molecular clock, one hundred million iterations, sampling model parameters every 20,000 iterations with 10% burn-in. We applied a mutation rate of 1.57 × 10^–7^ sub/site/generation, which is the mtDNA evolutionary rate of *Caenorhabditis elegans* [80], and a generation time of 40 days for the two pinworm species [54, 81]. For the host, we used a 1.25 × 10^–7^ sub/site/generation rate was, which is the adapted primate cyt*-b* evolutionary rate with a howler monkey generation time of 5 years [82, 83]. Plots and the performance of the MCMC process were visualized in Tracer v 1.5. [84].

The genealogical relationships between haplotypes were determined by unrooted median joining networks in Network v.5 [85] for each pinworm species and their primate host. When resulting networks were too complex, we ran a maximum parsimony post-processing to visualize the most parsimonious tree [86]. We tested a codivergence scenario between host and parasite linages using the ParaFit approach [87]. Bayesian maximum clade credibility trees of howler monkeys and *Trypanoxyuris minutus* haplotypes were built in MrBayes v.3.2.2 [88] and the CIPRES Science Gateway [89], including two simultaneous MCMC runs, each for four million generations, sampling trees every 4000 generations, and 25% burn-in. Patristic distance matrices of both host and parasite phylogenies were estimated with adephylo v.1.1 [90], and the ParaFit test was run in ape v.5 [91].

### Haplotype level analyses

Haplotype analyses were run only for *T. minutus* given the small sample size we had for *T. multilabiatus.*

To further examine howler-pinworm association patterns at the mtDNA level, we first identified (a) the host haplotypes infected by various *T. minutus* haplotypes, and (b) those infected by only one pinworm haplotype. Next, we estimated the genetic divergence (p-distance) between howler haplotypes that share pinworm haplotypes and between those infected by unique pinworm haplotypes. The same strategy was followed for the parasite, estimating genetic divergence between co-occurring haplotypes and those found in only one host haplotype. We performed Wilcoxon–Man Whitney tests in R to assess if the haplotypes that shared host/parasites were more like each other than to haplotypes harbouring or infecting distinct host/parasites. We also examined the relationship between susceptibility/infectivity and haplotype frequency and divergence by performing non-parametric correlation tests using the package ggpubr v.0.4 [92]. The latter enables evaluating if more common haplotypes were either more susceptible (howler haplotypes associated to many different pinworm haplotypes) or more infective (pinworm haplotypes found in various host haplotypes), and to identify if more divergent haplotypes had an infective/resistant advantage over genetically similar haplotypes. Finally, we expected similar host haplotypes to harbour similar pinworm haplotypes, thus we tested the correlation between the genetic distance of the host and their associated pinworm haplotypes.

## Supplementary Information

**Additional file 1.** Additional Tables S1-S5 and Figures S1–S4.

## Data Availability

Both primate host and parasites mtDNA sequences are available in GenBank (Additional file 1: Table S5); associated microsatellite genotypes of primate host are available at https://doi.org/10.5281/zenodo.4538731
