# Supplementary material for: Co-structure analysis and genetic associations reveal insights into pinworms (Trypanoxyuris) and primates (Alouatta palliata) microevolutionary dynamics
Source: BMC Ecol Evol. 2021 Oct 20;21:190. doi: 10.1186/s12862-021-01924-4 (PMC8527708; doi:10.1186/s12862-021-01924-4)
Supplement: Supplementary file 1 — Additional file 1. Additional Tables S1-S5 and Figures S1–S4. [file 12862_2021_1924_MOESM1_ESM.pdf]

Additional files

Table S1. Pairwise F_{ST} of *Alouatta palliata* mtDNA between geographic regions. Significant values are shaded in orange.

	TUX	SMT	UXP	CML	PCH
SMT	0.3722				
UXP	0.1299	0.1084			
CML	0.5664	0.4500	0.3215		
PCH	0.5215	0.2046	0.2136	0.3424	
AGA	0.4540	0.0521	0.1193	0.5894	0.2910

TUX: Los Tuxtlas, SMT: Santa Marta, UXP: Uxpanapa, CML: Comalcalco, PCH: Pichucalco, AGA: Agaltepec Island

Table S2. Pairwise F_{ST} of pinworm mtDNA between geographic regions. Under diagonal: *Trypanoxyuris minutus*; above diagonal: *T. multilabiatus* . Significant values are shaded in blue.

	TUX	SMT	UXP	CML	PCH	AGA
TUX		-----	-----	-----	-----	-----
SMT	-0.0176		-0.1374	1	0.6666	-0.2903
UXP	0.0122	0.0057		0.3782	0.3417	0.0226
CML	0.0786	0.0793	0.1663		0.5714	0.1960
PCH	0.0623	0.0747	0.1971	0.0088		0.2135
AGA	0.0068	0.0129	0.0443	0.0190	0.0203	

TUX: Los Tuxtlas, SMT: Santa Marta, UXP: Uxpanapa, CML: Comalcalco, PCH: Pichucalco, AGA: Agaltepec Island

Table S3. Pairwise F_{ST} of *Alouatta palliata* mtDNA between sampling locations. Significant values are shaded in orange.

	MP1	PL	MIP	VA	PA	MV	HL	ZA	PCH
PL	0.1957								
MIP	0.5995	0.3143							
VA	0.5248	0.36900	0.7363						
PA	0.3816	0.1457	0.3267	0.2500					
MV	0.2710	0.1358	0.5185	0.1111	0.0735				
HL	0.4540	0.3157	0.5602	0.1333	0.1868	0.0737			
ZA	0.4500	0.4007	0.7711	0.8400	0.4937	0.4538	0.6769		
PCH	0.5851	0.4397	0.6876	0.6790	0.4282	0.3257	0.5532	0.2243	
AGA	0.5215	0.4244	0.5797	0.3115	0.3250	0.0844	0.2910	0.4524	0.2601

Los Tuxtlas: Montepío (MP1); Santa Marta: Playa (PL), Mirador Pilapa (MIP), La Valentina (VA); Uxpanapa: Plan de Arroyo (PA), Murillo Vidal (MV); Comalcalco: Hacienda La Luz (HL), Archaeological Site (ZA); Pichucalco (PCH9; Agaltepec Island (AGA).

Table S4. Genetic diversity estimates for howler monkey (*Alouatta palliata*) and its pinworm populations (*Trypanoxyuris minutus* and *T. multilabiatus*) across regions and genetic clusters.

		Mt-DNA									Microsatellites		
		T. minutus			*T. multilabiatus*			*A. palliata*			*A. palliata*		
**Cluster**	**Region**	**n**	**Hd**	**π**	**n**	**Hd**	**π**	**n**	**Hd**	**π**	**Na**	**Ho**	**He**
WEST	TUX	11	0.98	0.0049	---	----	-----	8	0.75	0.0017	2.8	0.44	0.53
	SMT	25	0.97	0.0048	2	0.0	0.0	13	0.15	0.0013	4.0	0.35	0.56
	AGA	10	0.93	0.0051	5	0.9	0.0095	6	0.73	0.0009	2.6	0.37	0.49
	UXP	9	0.94	0.0044	5	0.70	0.0029	8	0.75	0.0021	3.3	0.39	0.55
	**subtotal**	**55**	**0.97**	**0.0049**	**12**	**0.68**	**0.0053**	**35**	**0.68**	**0.0019**	**5.0**	**0.37**	**0.57**
EAST	CML	20	0.98	0.0047	2	0.0	0.0	13	0.60	0.0007	3.8	0.38	0.56
	PCH	14	0.99	0.0043	2	1.0	0.0036	10	0.64	0.0011	3.9	0.38	0.49
	**subtotal**	**34**	**0.99**	**0.0045**	**4**	**0.83**	**0.0034**	**23**	**0.79**	**0.0011**	**4.6**	**0.38**	**0.54**
Total		89	0.98	0.0049	16	0.81	0.0056	58	0.79	0.0019	5.4	0.39	0.57

Table S5. GenBank accession numbers of the different mtDNA haplotypes obtained in this study for host and parasites, and the associated ID number of the host microsatellite genotype information available at https://doi.org/10.5281/zenodo.4538731

	mtDNA GenBank accession numbers			
**Haplotype**	***T. minutus***	***T. multilabiatus***	***A. palliata***	**Host genotype ID**
1	MF379155	KU285488	MW452904	E105
2	MF379159	OK381692	MW452899	E108, E118, E141, E129
3	MF379156	OK381693	MW452905	E139, E140
4	MF379158	OK381694	MW452906	E142
5	MF379205	OK381690	MW452908	E25
6	MF379151	KU285489	MW452910	E84
7	MF379211	KU285487	MW452911	E85, E70
8	MF379152	OK381691	MW452897	E3, E24, E26, E41, E79, E100, E20, E83, E67, E8, E7, E63, E4, E14, E55, E61, E45, E29, E39, E43
9	MF379202		MW452909	E6
10	MF379157		MW452902	E35
11	MF379221		MW452921	E94
12	MF379223		OK423769	E95
13	MF379226		MW452903	E31, E22, E57, E58, E44, E53
14	MF379227		MW452901	E78, E19, E18, E51, E9, E23, E54
15	MF379228		MW452900	E1
16	MF379224		OK423770	E60
17	MF379212		MW452907	E32, E36, E21, E47
18	MF379214		MW452896	E10, E27
19	MF379218		MW452898	E40
20	MF379219			
21	MF379201			
22	MF379203			
23	MF379204			
24	MF379206			
25	MF379193			
26	MF379195			
27	MF379197			
28	MF379199			
29	MF379200			
30	MF379235			
31	MF379229			
32	MF379230			
33	MF379236			
34	MF379234			
35	MF379233			
36	MF379231			
37	KU285482			
38	MF379232			
39	MF379238			
40	MF379240			
41	MF379241			
42	MF379243			
43	MF379245			
44	MF379107			
45	MF379248			

Table S5. Continue

	mtDNA GenBank accession numbers			
**Haplotype**	***T. minutus***	***T. multilabiatus***	***A. palliata***	**Host genotype ID**
46	MF379255			
47	MF379250			
48	MF379252			
49	MF379253			
50	MF379249			
51	MF379257			
52	MF379251			
53	MF379259			
54	MF379260			
55	MF379131			
56	KU285484			
57	MF379139			
58	MF379135			
59	MF379136			

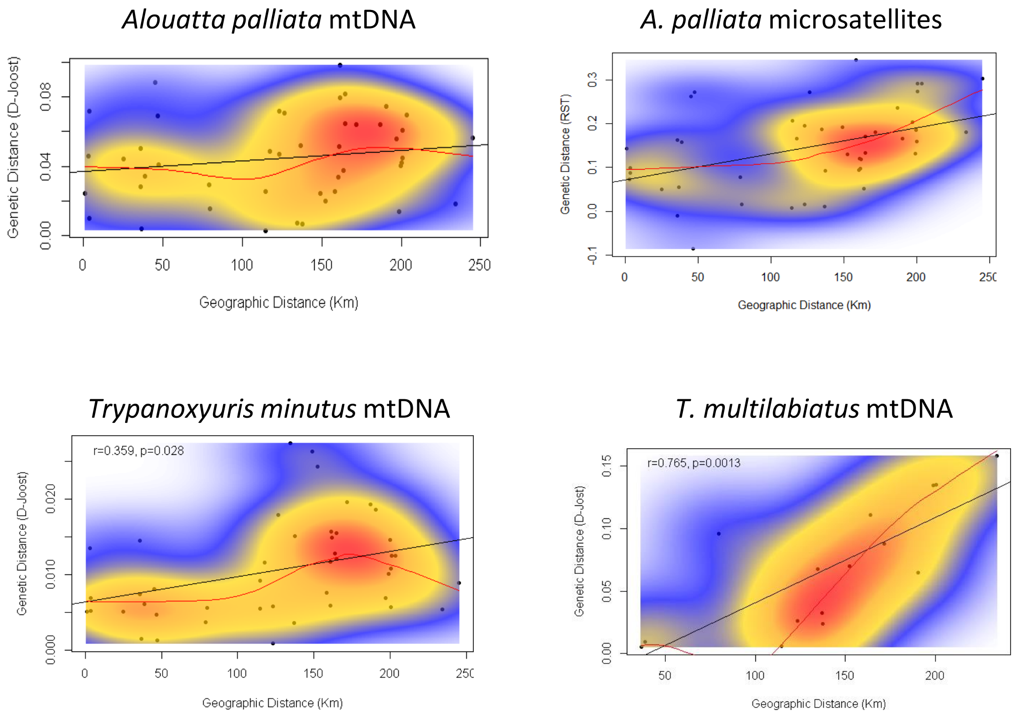

	D-Jost	Hedrick G´$_{ST}$	Edwards	F_{ST}
A. palliata cyt-b	r = 0.17 p = 0.18	r = 0.21 p = 0.11	r = 0.11 p = 0.28	r = 0.22 p = 0.1
A. palliata microsatellites	r = 0.07 p = 0.30	r = 0.11 p = 0.27	r = 0.17 p = 0.15	r = 0.44[◊] p = 0.004
T. minutus	r = 0.36 p = 0.03	r = 0.38 p = 0.02	r = 0.48 p = 0.006	r = 0.09 p = 0.29
T. multilabiatus	r = 0.77 p = 0.001	r = 0.54 p = 0.03	r = 0.68 p = 0.001	r = 0.34 p = 0.07

[◊]Estimated with R_{ST}

Figure S1. Mantel test results for isolation by distance patterns for howler monkeys (*Alouatta palliata*) and pinworms (*Trypanoxyuris minutus* and *T. multilabiatus*)

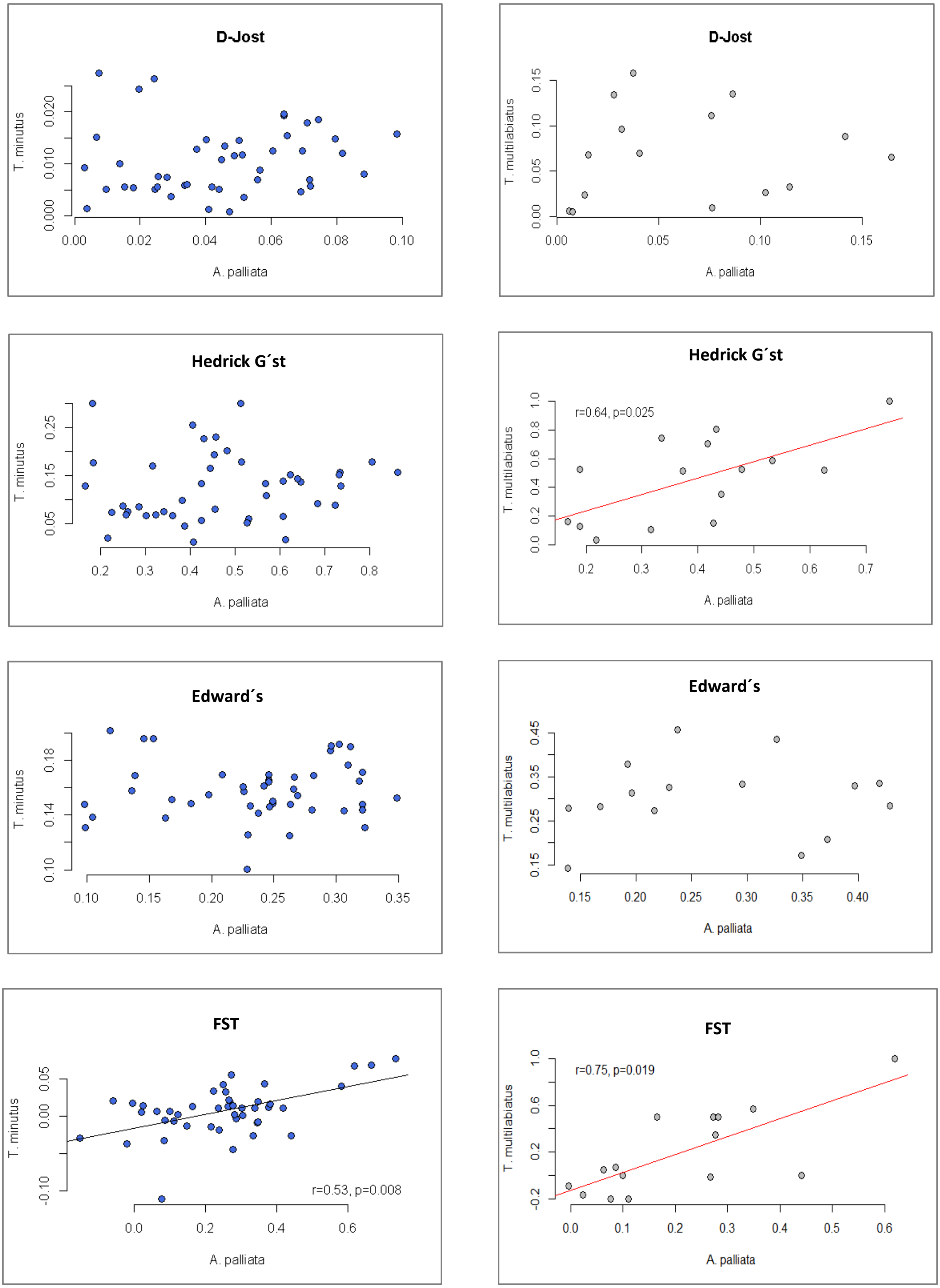

Figure S2. Correlations between host pairwise distance and those of its pinworm parasites. Left: *Trypanoxyuris minutus;* right: *T. multilabiatus*

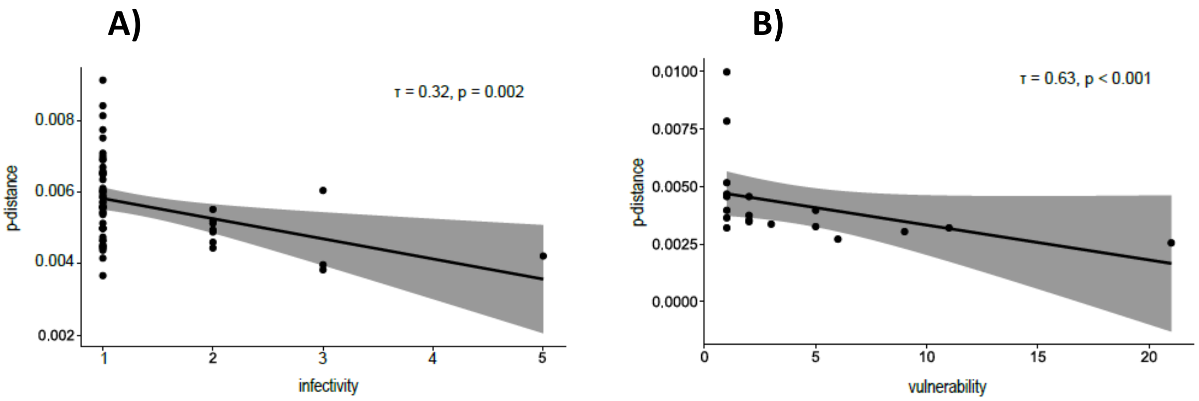

Figure S3. Kendall correlations between haplotype p-distance and A) *Trypanoxyuris minutus* haplotype infectivity, or B) *Alouatta palliata* haplotype vulnerability.

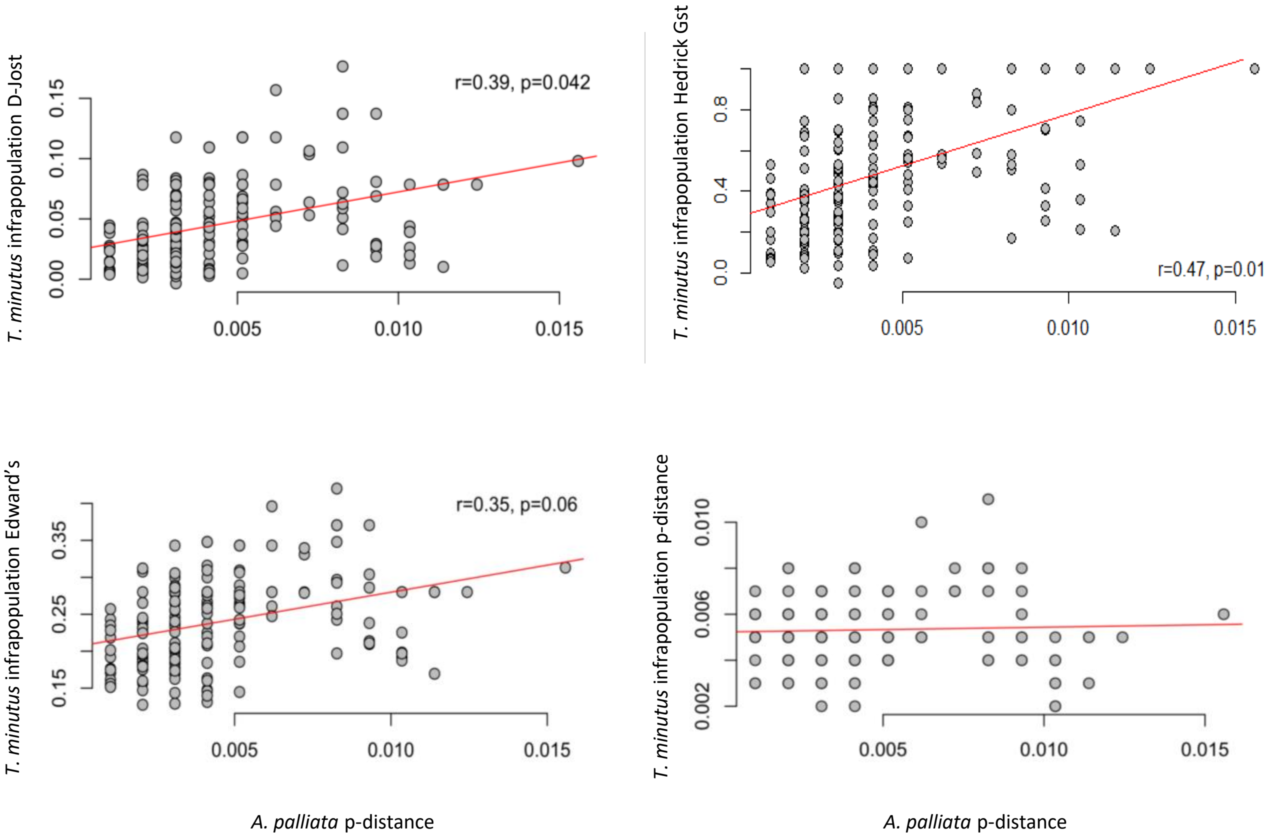

Figure S4. Correlations between host haplotype pairwise p-distance and the genetic distance of their associated pinworm infrapopulations haplotypes
